# Spectrum of Chromosomal Abnormalities in Abortus and Medically Terminated Fetal Samples From a Tertiary Care Center in the Sub-Himalayan Region of North India

**DOI:** 10.7759/cureus.105319

**Published:** 2026-03-16

**Authors:** Rashmi Malhotra, Mohit Jadli, Anupama Bahadur, Mukund Vatsa, Royana Singh, Jaya Chaturvedi, Yogendra P Mathuria, Bharti Jakhar, Mukesh Singla, Nilotpal Chowdhury

**Affiliations:** 1 Anatomy, All India Institute of Medical Sciences, Rishikesh, Rishikesh, IND; 2 Molecular Oncology, All India Institute of medical sciences, Rishikesh, Rishikesh, IND; 3 Obstetrics and Gynecology, All India Institute of Medical Sciences, Rishikesh, Rishikesh, IND; 4 Anatomy, Institute of Medical Sciences (IMS) Banaras Hindu University (BHU), Varanasi, IND; 5 Microbiology, All India Institute of Medical Sciences, Rishikesh, Rishikesh, IND; 6 Pathology, All India Institute of Medical Sciences, Rishikesh, Rishikesh, IND

**Keywords:** abortus, aneuploidy, chromosomal abnormalities, fetal anomalies, genetic counselling, pregnancy loss, qf-pcr, trisomy 21

## Abstract

Background: Chromosomal abnormalities are a major cause of spontaneous abortions and medically terminated pregnancies. Molecular characterization of abortus tissue plays a critical role in establishing etiological diagnosis, enabling accurate genetic counselling and recurrence risk assessment. However, region-specific molecular data from the sub-Himalayan population remain limited.

Objective: To evaluate the spectrum and frequency of chromosomal abnormalities in spontaneous abortus and medically terminated fetal samples using molecular genetic techniques at a tertiary care center in North India.

Methodology: This cross-sectional observational study included 39 fetal samples obtained following spontaneous abortion or medically indicated termination of pregnancy between August 2022 and February 2026 at a tertiary care referral center serving the sub-Himalayan region of North India. Following detailed gross examination and dissection, genomic DNA was extracted from fetal tissue and analyzed using the Devyser Extended Aneuploidy quantitative fluorescence polymerase chain reaction (QF-PCR) assay targeting chromosomes 13, 15, 16, 18, 21, X, and Y. The samples underwent additional evaluation using Sanger sequencing for confirmation and further characterization of detected chromosomal abnormalities. Molecular findings were correlated with clinical characteristics and prenatal imaging data.

Results: Chromosomal abnormalities were identified in 10 of 39 cases (25.6%). Trisomy 21 was the most frequently detected abnormality (7, 17.9%), followed by trisomy 18 (1, 2.6%). Trisomy 22 was identified in one case (2.6%) and monosomy X in one case (2.6%). Central nervous system anomalies constituted the most common structural abnormalities and were frequently observed in fetuses with chromosomal defects. The majority of cases (29, 74.4%) demonstrated a normal euploid genotype, suggesting potential contribution of non-chromosomal or maternal factors.

Conclusions: Chromosomal abnormalities contribute significantly to pregnancy loss in this study, with autosomal trisomies representing the predominant genetic findings. QF-PCR provides a rapid and reliable diagnostic approach for detecting common aneuploidies in abortus tissue. Routine incorporation of molecular testing may enhance etiological clarification and improve genetic counselling and reproductive planning in affected couples.

## Introduction

Pregnancy loss represents one of the most common adverse outcomes in human reproduction and continues to pose a major clinical and public health challenge worldwide. Congenital anomalies and chromosomal abnormalities constitute significant contributors to spontaneous abortions, stillbirths, and medically indicated termination of pregnancies. Epidemiological studies from different regions have reported that congenital malformations account for a considerable proportion of perinatal morbidity and mortality, particularly in developing countries where surveillance systems and access to advanced prenatal diagnostic technologies may be limited [1,2]. Pregnancy loss not only has biological implications but also imposes substantial psychological, emotional, and social stress on affected couples, often resulting in long-term psychological consequences and impaired reproductive confidence [3-6].

The global burden of congenital anomalies has been recognized as an important public health issue by international health organizations. The World Health Organization (WHO) has emphasized the need for improved surveillance, early detection, and preventive strategies to reduce the incidence and impact of birth defects worldwide [7,8]. Prenatal diagnostic techniques, particularly second-trimester ultrasonography, have become essential tools for identifying structural fetal abnormalities and guiding obstetric management decisions [9]. When severe fetal anomalies incompatible with life or associated with significant morbidity are detected, medical termination of pregnancy may be considered. In such cases, detailed post-termination examination of fetal tissue can provide valuable insights into the underlying etiology of pregnancy loss and facilitate accurate recurrence risk assessment for future pregnancies [10].

Fetal autopsy and systematic morphological evaluation remain important components of the diagnostic workup in cases of pregnancy loss and congenital anomalies. Several studies have demonstrated that postmortem examination can confirm prenatal imaging findings and may also reveal additional anomalies not detected during antenatal screening [11-13]. Detailed pathological examination of fetal and placental tissues contributes significantly to understanding the mechanisms underlying fetal demise and congenital malformations [14]. These investigations are particularly valuable when combined with molecular genetic techniques, which can identify chromosomal abnormalities and genetic defects responsible for abnormal fetal development.

Chromosomal abnormalities represent one of the most important genetic causes of pregnancy loss. Among chromosomal abnormalities, aneuploidies involving chromosomes 13, 18, and 21, as well as sex chromosome abnormalities, are among the most frequently encountered in products of conception. Trisomy 21 (Down syndrome) is the most common viable autosomal trisomy and is associated with characteristic craniofacial features, congenital heart defects, and developmental delay. Trisomy 18 (Edwards syndrome) is associated with severe growth restriction, cardiac anomalies, and high perinatal mortality. Midline defects, central nervous system anomalies, and craniofacial malformations typically characterize trisomy 13 (Patau syndrome). In addition, sex chromosome abnormalities, particularly monosomy X (Turner syndrome), are commonly detected in spontaneous abortions and may present with cystic hygroma, hydrops fetalis, or fetal growth restriction. Understanding the spectrum of these chromosomal abnormalities is essential for accurate etiological diagnosis and genetic counselling. Autosomal trisomies, monosomies, and other chromosomal aneuploidies are frequently detected in products of conception from spontaneous abortions and medically terminated pregnancies. Several studies from India and other countries have reported varying prevalence of congenital anomalies and chromosomal abnormalities depending on geographical, environmental, and maternal health factors [15]. Such regional variations highlight the need for population-specific studies to better understand the spectrum and underlying causes of fetal anomalies.

Advances in molecular cytogenetic techniques have significantly improved the detection of chromosomal abnormalities in fetal tissue. Quantitative fluorescence polymerase chain reaction (QF-PCR) has emerged as a rapid and reliable method for detecting common aneuploidies involving chromosomes 13, 18, 21, and sex chromosomes. Recent studies have demonstrated the usefulness of QF-PCR and chromosomal microarray analysis in identifying chromosomal abnormalities in products of conception obtained from spontaneous miscarriages [16-18]. These molecular approaches offer advantages over conventional cytogenetic techniques, including faster turnaround time and improved diagnostic yield.

Despite the availability of advanced molecular diagnostic techniques, region-specific data integrating fetal morphological assessment with molecular genetic analysis remain limited in many parts of India, particularly in the sub-Himalayan region. Environmental influences, maternal health conditions, and genetic background may contribute to regional differences in the pattern of congenital anomalies and pregnancy loss. Therefore, studies focusing on specific populations are essential to improve understanding of the underlying etiological factors and to guide preventive and diagnostic strategies.

The present study was undertaken to evaluate the spectrum and frequency of chromosomal abnormalities in spontaneous abortus and medically terminated fetal samples using molecular genetic techniques at a tertiary care center in North India.

## Materials and methods

This cross-sectional observational study was conducted at the All India Institute of Medical Sciences (AIIMS), Rishikesh, a tertiary care referral center catering to the sub-Himalayan population of Uttarakhand and adjoining regions of Western Uttar Pradesh, over the period from August 2022 to February 2026. Fetal samples were collected following approval from the Institutional Ethics Committee (IEC), AIIMS Rishikesh (Approval No. AIIMS/IEC/22/435). Analysis and structured data evaluation were undertaken after obtaining additional approval from the Institutional Ethics Committee, AIIMS Rishikesh (Letter No. AIIMS/IEC/25/240). Written informed consent was obtained from parents before inclusion in the study in accordance with the principles of the Declaration of Helsinki.

A total of 39 aborted fetuses were included in the study using convenience sampling. All eligible cases received at the institution following spontaneous abortion or legal termination of pregnancy were considered for inclusion. Fetuses that were structurally intact and had adequate tissue preservation were included in the study. Macerated fetuses and samples obtained from non-consenting parents were excluded. Following expulsion or evacuation, fetal tissue was collected under aseptic precautions from the Department of Obstetrics and Gynecology after obtaining written parental consent. Clinical data were obtained from hospital records and included maternal age, obstetric history, gestational age at pregnancy loss, antenatal ultrasonography findings, and associated maternal medical or metabolic conditions such as hypothyroidism, hypertensive disorders, gestational diabetes mellitus, anemia, and other systemic illnesses. 

The fetus was transported to the Department of Anatomy in normal saline for detailed examination. Fetal parameters, including gestational age, sex where identifiable, gross morphological features, and the presence of congenital anomalies, were documented. Placental examination was performed in cases where placental tissue was available and adequately preserved for pathological evaluation. Representative tissue samples were selected based on preservation and suitability for molecular analysis and were transferred to the Multidisciplinary Research Unit (MRU) of the institute for further processing.

Genomic DNA was extracted from fetal tissue using a standard DNA isolation protocol with the QIAamp DNA Mini Kit (Qiagen, Hilden, Germany). The quality and purity of the extracted DNA were assessed using the Qubit 4 Fluorometer (Invitrogen, Thermo Fisher Scientific, Waltham, MA) before molecular analysis to ensure adequate yield and integrity for downstream testing.

Rapid aneuploidy analysis was performed using the Devyser Extend v2 quantitative fluorescent polymerase chain reaction (QF-PCR) kit (Devyser AB, Stockholm, Sweden) following the manufacturer’s protocol. This assay targeted chromosomes 13, 15, 16, 18, 21, X, and Y for the detection of common chromosomal aneuploidies. PCR amplification products were separated by capillary electrophoresis on an ABI 3500 Genetic Analyzer (Applied Biosystems, Thermo Fisher Scientific, Waltham, MA). Fragment sizing was performed using the 560 Sizer Orange internal size standard compatible with the Devyser Extend QF-PCR kit. The resulting electropherograms were analyzed using GeneMapper Software version 5.0 (Applied Biosystems, Thermo Fisher Scientific) for allele calling and dosage interpretation.

All samples were tested for aneuploidies involving chromosomes 13, 18, 21, and the sex chromosomes. In addition, 28 of the 39 samples were further evaluated for aneuploidies involving chromosomes 15, 16, and 22 using the extended QF-PCR panel. Selected samples with abnormal molecular findings or requiring further characterization underwent additional evaluation using Sanger sequencing for confirmation and targeted genetic analysis.

Molecular findings were correlated with prenatal diagnostic investigations, including ultrasonography and amniocentesis results, wherever available. All collected data were compiled and analyzed using SPSS version 27 (IBM Corp., Armonk, NY). Descriptive statistics, including frequencies, percentages, mean, and standard deviation, were calculated for demographic, clinical, and molecular variables.

## Results

**Figure 1:**
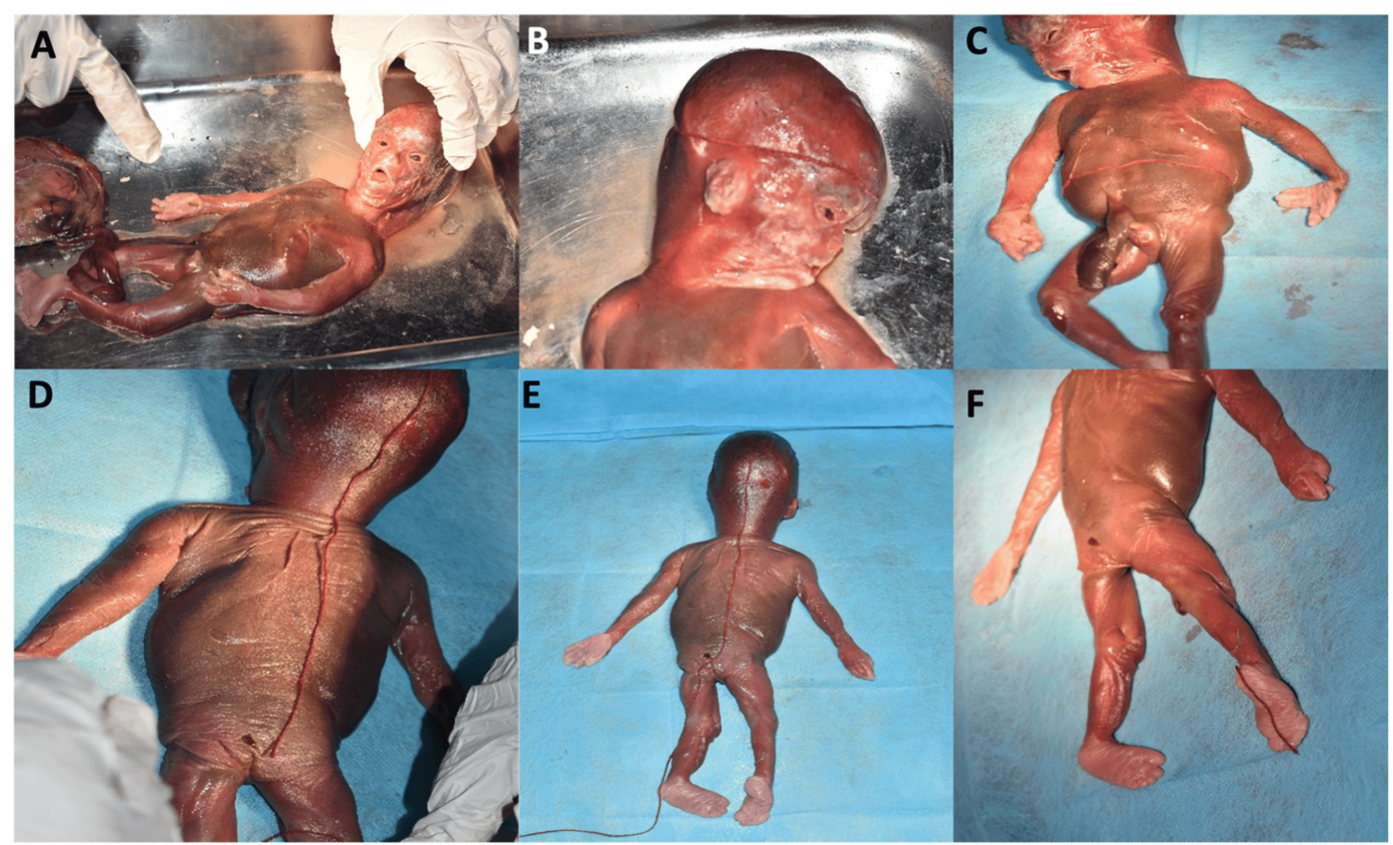
External morphometric assessment of a fetal specimen. (A) Ventral aspect of the fetus demonstrating overall external morphology before measurement. (B) Measurement of head circumference for the assessment of cranial growth parameters. (C) Measurement of abdominal circumference as part of the external fetal morphometric evaluation. (D) Measurement of crown-rump length (CRL), used for the estimation of fetal growth and correlation with gestational age. (E) Measurement of crown-heel length (CHL), representing the total longitudinal body length. (F) Measurement of foot length, used as an additional anthropometric parameter for gestational age estimation.

A total of 39 abortus and medically terminated fetal samples were included in the present study. The baseline demographic and fetal characteristics of the study population are summarized in Table 1. Pregnancy loss was observed across a gestational age range of 10-38 weeks, with the second trimester accounting for the majority of cases (approximately 59%). Male fetuses constituted 21 (53.8%) cases, while female fetuses accounted for 14 (35.9%) cases. In four cases (10.3%), fetal sex could not be determined due to early gestational age or inadequate tissue preservation. The mean fetal weight was 735 ± 560 g, with a range of 60-2,900 g. External morphometric assessment of representative fetal specimens, including measurements of head circumference, abdominal circumference, crown-rump length, crown-heel length, and foot length, is illustrated in Figure 1.

**Table 1:** Baseline characteristics of abortus and medically terminated fetal samples included in the study (n = 39). Data represent demographic and fetal parameters of the study cohort. Percentages were calculated based on the total sample size (*n* = 39).

Parameter	Observation
Total number of fetal samples	39
Gestational age range (weeks)	10-38
Predominant gestational period	Second trimester (59%)
Male fetuses, *n* (%)	21 (53.8%)
Female fetuses, *n* (%)	14 (35.9%)
Sex not identifiable	4 (10.3%)
Mean fetal weight (g)	735 ± 560
Fetal weight range (g)	60-2,900

Spectrum of congenital anomalies

**Figure 2:**
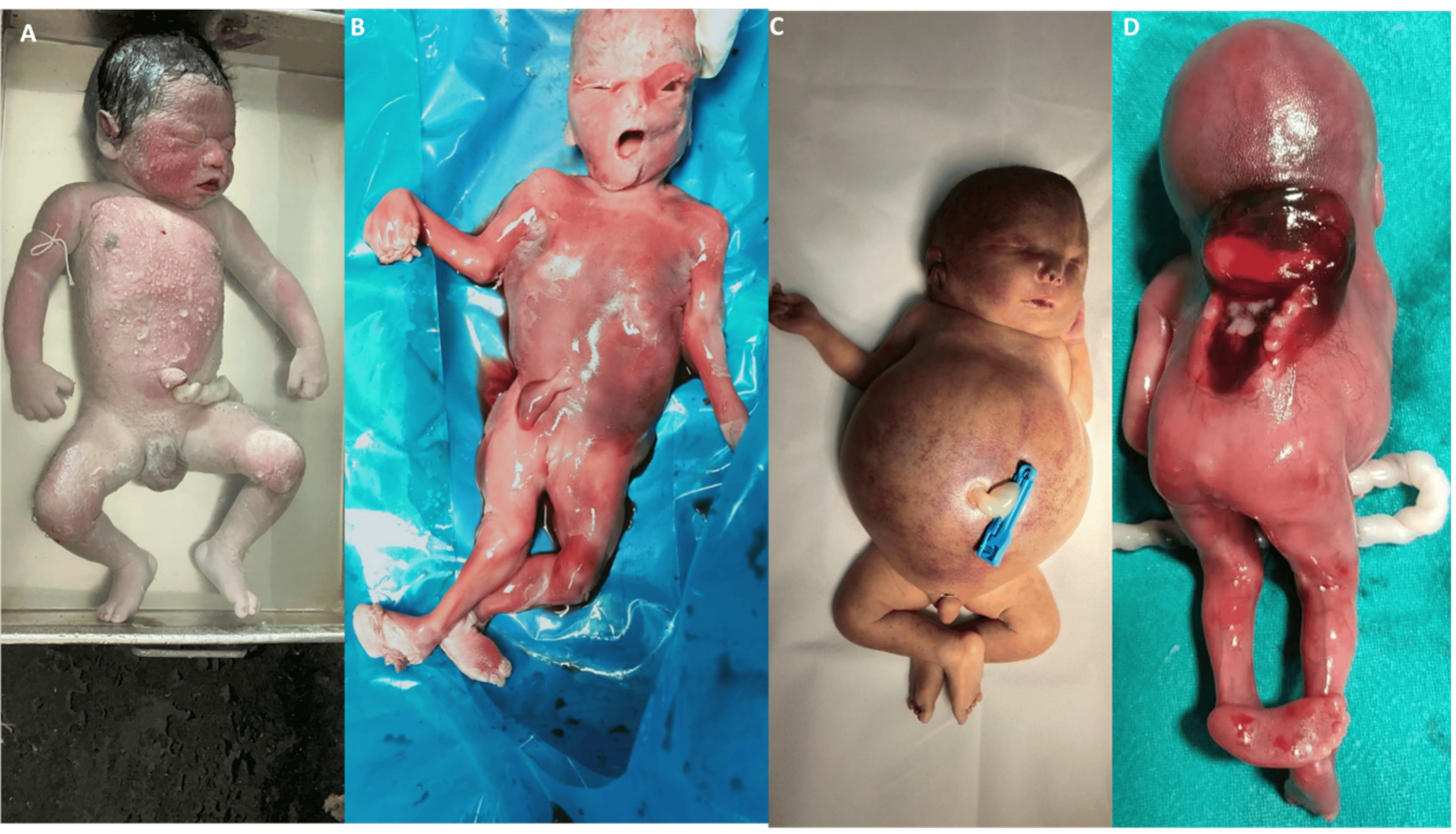
Gross morphological spectrum of chromosomal and congenital abnormalities observed in fetal samples. (A) Fetus with features consistent with trisomy 21 (Down syndrome), showing characteristic dysmorphic facial features and generalized soft tissue edema. (B) Fetus demonstrating phenotypic features suggestive of trisomy 18 (Edwards syndrome), including growth restriction and abnormal limb posture. (C) Fetus with hydrops fetalis, characterized by marked generalized edema and abdominal distension. (D) Fetus demonstrating a severe craniofacial malformation with a major anterior facial defect, representing a complex congenital structural anomaly; definitive chromosomal correlation requires molecular confirmation.

The distribution of congenital anomalies identified during gross examination of the fetal specimens is presented in Table 2. Central nervous system (CNS) anomalies constituted the most frequently observed category (14, 34.1%), including hydrocephalus, neural tube defects, and cranial malformations. Cardiovascular anomalies were the second most common group (6, 14.6%), followed by renal and genitourinary anomalies (5, 12.2%). Skeletal deformities were noted in four cases (9.8%), craniofacial anomalies in three cases (7.3%), and multisystem congenital anomalies in five cases (12.2%). Hydrops fetalis was observed in four cases (9.8%), characterized by generalized fetal edema and marked developmental compromise. In several fetuses, anomalies involved more than one organ system. Representative examples of the phenotypic spectrum of congenital anomalies observed in the study cohort are demonstrated in Figure 2.

**Table 2:** Spectrum of congenital anomalies observed in fetal samples (n = 39). Distribution of structural anomalies identified during gross fetal examination. Some fetuses demonstrated involvement of more than one organ system. Neural tube defects such as anencephaly and spina bifida were included under central nervous system anomalies.

Category of anomaly	Representative findings	Number of cases (*n*)	Percentage (%)
Central nervous system	Hydrocephalus, neural tube defects, cranial anomalies	14	34.1
Cardiovascular	Structural cardiac defects	6	14.6
Renal/genitourinary	Renal anomalies	5	12.2
Skeletal	Limb deformities, skeletal dysplasia	4	9.8
Craniofacial	Facial dysmorphism, cleft anomalies	3	7.3
Multisystem anomalies	Multiple congenital anomalies	5	12.2
Hydrops fetalis	Generalized fetal edema	4	9.8

Chromosomal abnormalities

Molecular genetic analysis using QF-PCR and subsequent Sanger sequencing was successfully performed in all 39 cases. The spectrum of chromosomal abnormalities detected in the study population is summarized in Table 3. Chromosomal abnormalities were identified in 10 of the 39 cases (25.6%). Trisomy 21 (Down syndrome) was the most frequently detected abnormality, observed in 7 cases (17.9%), including three cases showing allelic peak ratios, suggestive of mosaic trisomy 21 on QF-PCR analysis. Trisomy 18 was identified in one case (2.6%), while trisomy 22 was detected in one case (2.6%). Monosomy X (Turner syndrome) was also identified in one case (2.6%). The remaining 29 cases (74.4%) demonstrated a normal euploid chromosomal constitution without evidence of aneuploidy.

**Table 3:** Chromosomal abnormalities detected by QF-PCR analysis (n = 39). Chromosomal abnormalities were detected using QF-PCR targeting chromosomes 13, 15, 16, 18, 21, X, and Y. Percentages were calculated relative to the total sample size (n = 39). QF-PCR, quantitative fluorescent polymerase chain reaction

Chromosomal finding	Number of cases (n)	Percentage (%)
Trisomy 21 (Down syndrome)	7	17.9
Full trisomy 21	4	10.3
Mosaic trisomy 21	3	7.7
Trisomy 18 (Edwards syndrome)	1	2.6
Trisomy 22	1	2.6
Monosomy X (Turner syndrome)	1	2.6
Normal euploid	29	74.4

Chromosomal abnormalities were frequently associated with major structural anomalies, particularly those involving the CNS and cardiovascular system. Representative electropherogram output obtained from QF-PCR analysis demonstrating the characteristic allele peak pattern consistent with trisomy 21 (Down syndrome) is illustrated inFigure 3.

**Figure 3:**
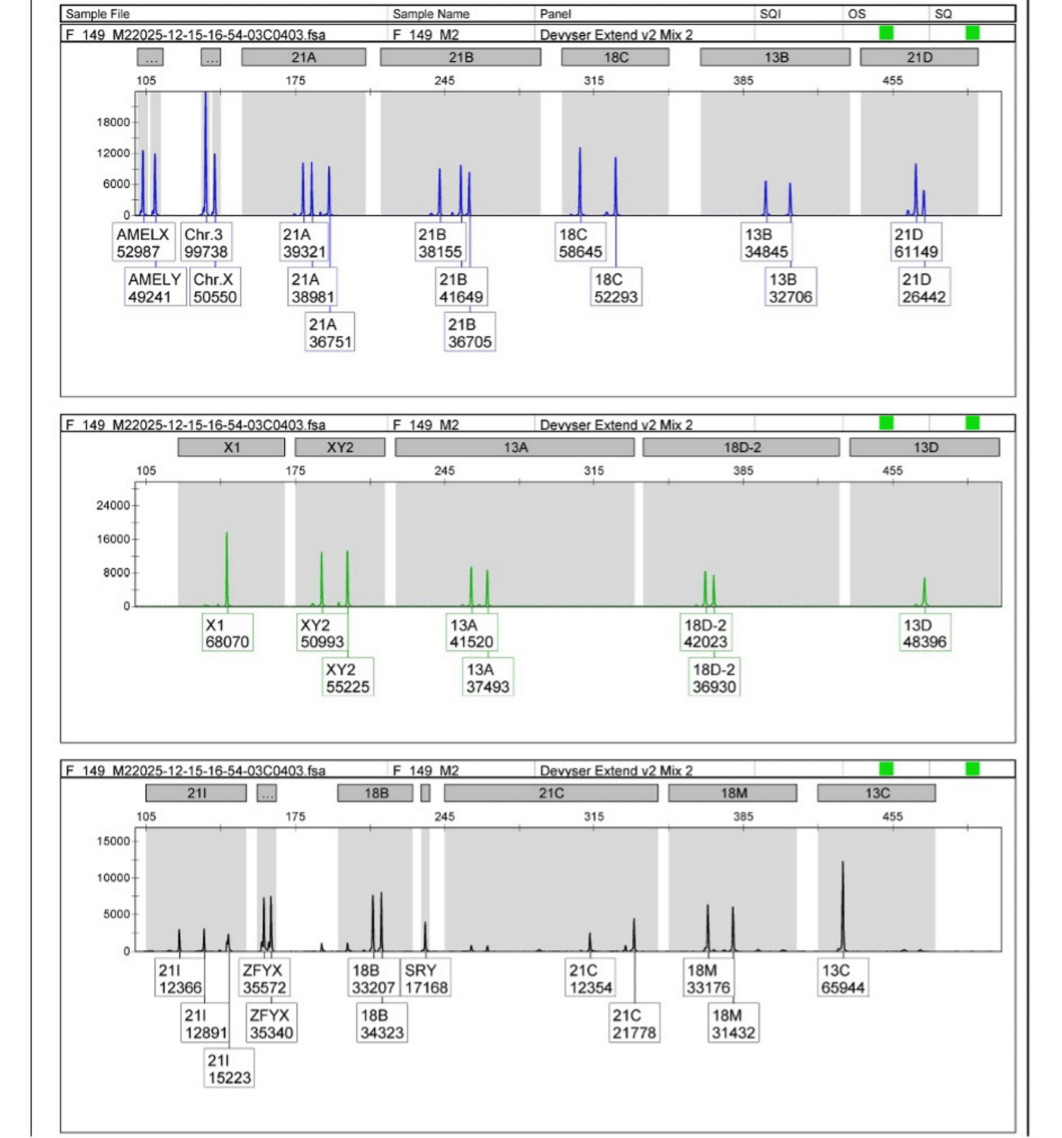
Representative QF-PCR electropherogram demonstrating chromosomal aneuploidy analysis. Representative electropherogram generated using quantitative fluorescent polymerase chain reaction (QF-PCR) with the Devyser Extended Aneuploidy panel. Fluorescent short tandem repeat (STR) markers corresponding to chromosomes 13, 18, 21, X, and Y are shown, illustrating allele peak patterns used for the detection of chromosomal dosage abnormalities. Peak number and relative signal intensity were analyzed to determine chromosomal copy number and identify aneuploidies.

Maternal clinical and metabolic conditions

Maternal clinical and metabolic conditions documented in the study cohort are summarized in Table 4. Hypothyroidism was the most frequently observed maternal condition (9, 23.1%), followed by hypertensive disorders (5, 12.8%) and oligohydramnios (5, 12.8%). Gestational diabetes mellitus and anemia were each observed in four (10.3%) cases, while other systemic illnesses were documented in three (7.8%) cases. In nine pregnancies (23.1%), no major maternal medical disorder was identified. These maternal conditions were frequently associated with structural fetal anomalies and adverse pregnancy outcomes, including intrauterine growth restriction and intrauterine fetal demise.

**Table 4:** Maternal clinical and metabolic conditions associated with pregnancy loss (n = 39). Maternal clinical and metabolic conditions documented during pregnancy. Associations represent observed clinical correlations and do not imply causation. CNS, central nervous system

Maternal condition	*n* (%)	Common fetal outcome observed
Hypothyroidism	9 (23.1%)	CNS anomalies, chromosomal abnormalities
Hypertensive disorders	5 (12.8%)	Growth restriction, intrauterine fetal demise
Gestational diabetes mellitus	4 (10.3%)	Cardiac anomalies
Anemia	4 (10.3%)	Low fetal weight
Oligohydramnios	5 (12.8%)	Renal anomalies
Other systemic illnesses	3 (7.8%)	Multisystem anomalies
No major maternal disorder	9 (23.1%)	Isolated anomalies/normal genetics

Integrated clinicopathological correlation

An integrated analysis of fetal morphology, chromosomal findings, and maternal clinical parameters is presented in Table 5. CNS anomalies and intrauterine growth restriction showed association with trisomy 21 and trisomy 18, particularly in cases with maternal hypothyroidism. Cardiac anomalies were frequently observed in fetuses with trisomy 21 and maternal gestational diabetes mellitus. Hydrops fetalis was observed in both chromosomally abnormal and chromosomally normal fetuses, suggesting a multifactorial etiology. Renal anomalies were commonly associated with oligohydramnios, while multiple congenital anomalies were observed in association with both chromosomal abnormalities and maternal systemic illnesses.

**Table 5:** Integrated clinicopathological correlation between fetal anomalies, chromosomal abnormalities, and maternal conditions. Integrated overview demonstrating clinicopathological associations identified in the study cohort. CNS, central nervous system

Fetal anomaly	Chromosomal abnormality	Associated maternal condition
CNS anomalies	Trisomy 21/Trisomy 18	Hypothyroidism
Cardiac anomalies	Trisomy 21	Gestational diabetes
Hydrops fetalis	Aneuploidy/normal	Maternal metabolic disorders
Renal anomalies	Normal aneuploidy	Oligohydramnios
Multiple congenital anomalies	Trisomy 18/normal	Systemic maternal illness

Overall, the findings of the present study demonstrate that chromosomal abnormalities contribute significantly to pregnancy loss in this population, with autosomal trisomies representing the predominant genetic abnormalities. 

**Figure 4:**
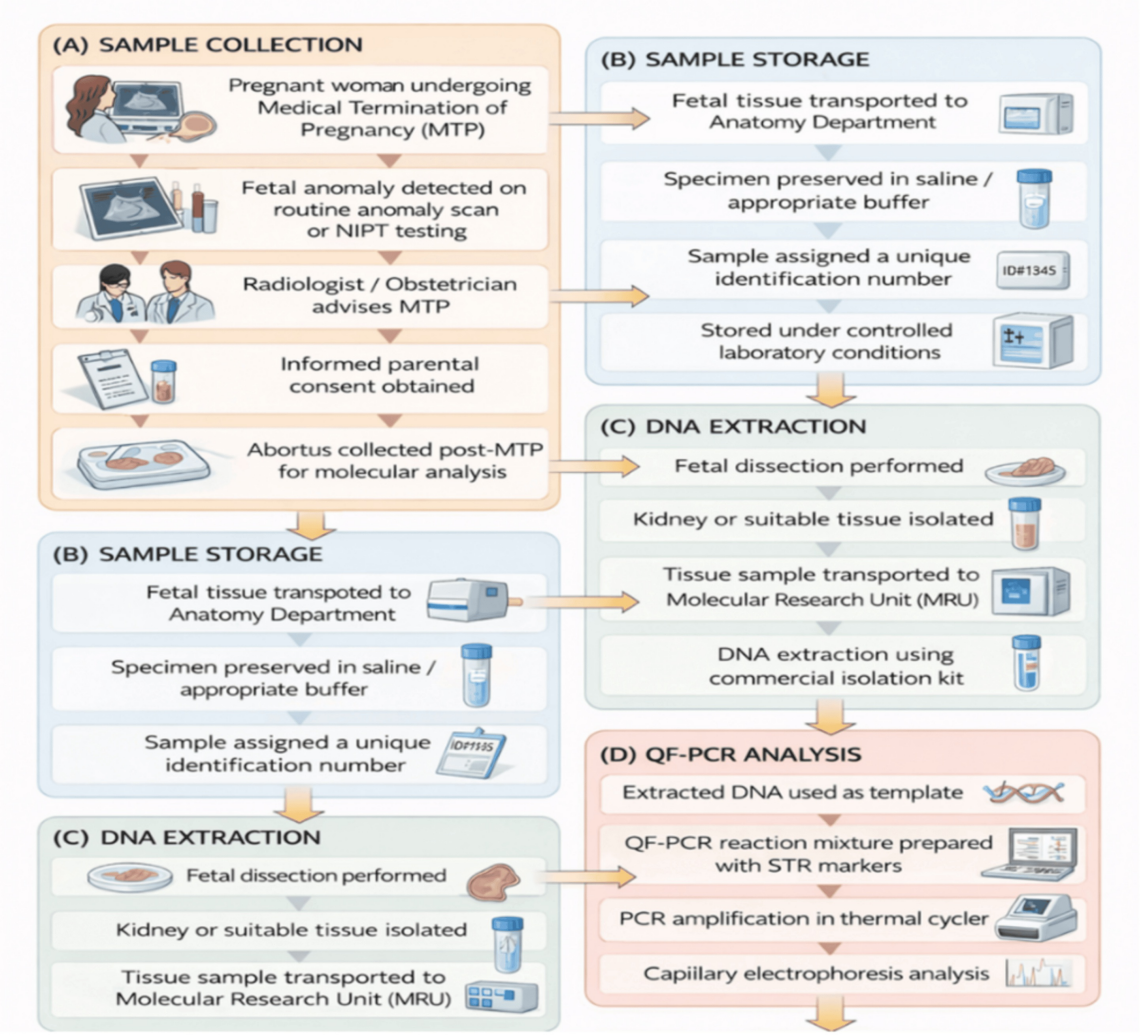
Methodological workflow of fetal tissue collection and molecular genetic analysis. Schematic representation of the methodological workflow followed in the present study for molecular evaluation of fetal tissue samples. (A) Sample collection: Pregnant women undergoing medical termination of pregnancy (MTP) following detection of fetal anomalies on routine anomaly scan or non-invasive prenatal testing (NIPT). After appropriate clinical evaluation and counselling by the obstetrician and radiologist, informed parental consent was obtained, and abortus specimens were collected for molecular analysis. (B) Sample storage: Fetal tissue specimens were transported to the Department of Anatomy, preserved in saline or appropriate buffer, assigned unique identification numbers, and stored under controlled laboratory conditions. (C) DNA extraction: Following fetal dissection, suitable tissue samples, such as kidney or other well-preserved tissues, were isolated and transported to the Multidisciplinary Research Unit (MRU), where genomic DNA was extracted using a commercial DNA isolation kit. (D) Quantitative fluorescent polymerase chain reaction (QF-PCR) analysis: Extracted DNA was used as a template for QF-PCR using short tandem repeat (STR) markers. PCR amplification was performed in a thermal cycler, and the amplified products were analyzed by capillary electrophoresis to detect chromosomal aneuploidies. Image credit: All authors.

The laboratory workflow involved in tissue processing, DNA extraction, and molecular analysis using QF-PCR is illustrated in Figure 4.

## Discussion

Pregnancy loss remains one of the most common adverse outcomes in human reproduction and continues to represent a significant challenge for clinicians and researchers alike. Congenital anomalies and chromosomal abnormalities are recognized as major contributors to spontaneous abortions, stillbirths, and medically indicated termination of pregnancy. Understanding the genetic and morphological basis of fetal anomalies is therefore essential for improving diagnostic accuracy, genetic counselling, and reproductive planning for affected couples. The present study evaluated the spectrum of chromosomal abnormalities in abortus and medically terminated fetal samples from a tertiary care center serving the sub-Himalayan population of North India, integrating fetal morphological examination with molecular genetic analysis.

In the present study, chromosomal abnormalities were detected in 10 (25.6%) of cases, with autosomal trisomies representing the predominant genetic findings. This observation is consistent with several previous studies that have reported chromosomal abnormalities as a major cause of pregnancy loss. Epidemiological studies have demonstrated that congenital anomalies and genetic defects contribute significantly to perinatal mortality and fetal morbidity across different populations [1,2]. In developing countries, the burden of congenital anomalies is particularly high due to limited access to early prenatal screening and advanced diagnostic facilities. Consequently, systematic evaluation of fetal tissue following pregnancy loss becomes an important tool for identifying underlying etiological factors.

Among the chromosomal abnormalities identified in the present study, trisomy 21 emerged as the most frequently detected abnormality, followed by trisomy 18 and trisomy 22, while monosomy X was identified in one case. These findings are broadly consistent with previous reports indicating that autosomal trisomies represent the most common chromosomal abnormalities associated with fetal anomalies and pregnancy loss. Studies examining products of conception have similarly reported trisomy 21, trisomy 18, and trisomy 13 among the most frequently encountered chromosomal abnormalities [16-18]. The predominance of autosomal trisomies in such cases is generally attributed to meiotic nondisjunction events during gametogenesis, particularly in maternal meiosis. Although the present study did not specifically investigate parental chromosomal factors, the findings align with established genetic mechanisms underlying fetal chromosomal abnormalities.

The detection of trisomy 21 in several cases within the present cohort also reflects the well-established association between this chromosomal abnormality and congenital anomalies. Down syndrome is known to be associated with a wide spectrum of structural abnormalities, including congenital heart defects, craniofacial dysmorphism, and developmental delay. Previous clinical and genetic studies have reported that fetuses with trisomy 21 often demonstrate structural anomalies detectable during prenatal imaging or postmortem examination [3]. The identification of trisomy 21 in the present study, therefore, reinforces the importance of molecular genetic testing in cases of fetal anomalies and pregnancy loss.

Structural anomalies identified during fetal examination in the present study involved multiple organ systems. CNS anomalies constituted the most frequently observed category, followed by cardiovascular, renal, skeletal, and craniofacial anomalies. The predominance of CNS malformations is consistent with previous studies of fetal autopsies and congenital anomaly surveillance programs. Neural tube defects, hydrocephalus, and other cranial malformations are among the most commonly reported congenital anomalies worldwide [2]. These abnormalities arise during early embryonic development, particularly during the period of neural tube closure in the first trimester, making them highly susceptible to genetic and environmental influences.

Cardiovascular anomalies formed the second most common group of congenital anomalies in the present study. Congenital heart defects are widely recognized as the most common congenital malformations encountered in clinical practice and frequently occur in association with chromosomal abnormalities. Numerous studies have demonstrated strong associations between congenital heart defects and chromosomal disorders such as trisomy 21, trisomy 18, and Turner syndrome [2]. The findings of the present study, therefore, support previously reported observations highlighting the genetic basis of many congenital cardiac anomalies.

Despite the detection of chromosomal abnormalities in a subset of cases, the majority of fetal samples in the present study demonstrated normal aneuploidy screening results, indicating that pregnancy loss is often multifactorial in origin. Several studies have emphasized that a large proportion of pregnancy losses occur in the absence of detectable chromosomal abnormalities. In such cases, other factors, including maternal metabolic disorders, placental insufficiency, infections, and environmental influences, may contribute to fetal demise [11-14]. The high proportion of chromosomally normal cases observed in the present study underscores the importance of evaluating maternal clinical conditions and environmental factors when investigating pregnancy loss.

The present study also examined maternal clinical and metabolic conditions associated with pregnancy loss. Hypothyroidism emerged as the most frequently observed maternal condition, followed by hypertensive disorders, oligohydramnios, gestational diabetes mellitus, and anemia. Maternal endocrine and metabolic disorders have been widely implicated in adverse pregnancy outcomes. For example, maternal thyroid dysfunction has been associated with impaired fetal neurodevelopment and increased risk of pregnancy complications, while gestational diabetes mellitus has been linked to congenital anomalies, particularly cardiovascular malformations [15]. Although causal relationships cannot be definitively established in the present study, the observed associations highlight the importance of comprehensive maternal evaluation in pregnancies complicated by fetal anomalies.

Another important aspect of the present study was the integration of fetal morphological examination with molecular genetic analysis. Fetal autopsy and systematic morphological assessment remain invaluable tools in the evaluation of congenital anomalies. Several studies have demonstrated that postmortem examination can confirm prenatal imaging findings and may also identify additional anomalies not detected during antenatal ultrasonography [10-13]. Detailed examination of fetal and placental tissues, therefore, plays a critical role in establishing accurate diagnoses and improving genetic counselling for affected families.

The use of QF-PCR in the present study provided a rapid and reliable method for detecting common chromosomal aneuploidies. QF-PCR has emerged as an effective alternative to conventional cytogenetic techniques due to its shorter turnaround time and relatively lower cost. Recent molecular studies have demonstrated that QF-PCR can accurately detect aneuploidies involving chromosomes 13, 18, 21, and sex chromosomes in products of conception [16-18]. The successful application of QF-PCR in the present study further supports its usefulness as a diagnostic tool in evaluating fetal chromosomal abnormalities, particularly in settings where conventional cytogenetic analysis may be limited by tissue viability or laboratory infrastructure.

The integration of molecular genetic testing with clinical and morphological evaluation provides a more comprehensive approach to understanding the etiology of pregnancy loss. In the present study, correlations between fetal anomalies, chromosomal abnormalities, and maternal clinical conditions were identified, suggesting that pregnancy loss often results from complex interactions between genetic and environmental factors. Such integrated clinicopathological analysis is essential for improving diagnostic accuracy and providing meaningful genetic counselling to affected couples.

Another important consideration highlighted by the present study is the need for region-specific data on congenital anomalies and pregnancy loss. Environmental exposures, maternal health conditions, and genetic background may vary considerably across different populations, potentially influencing the prevalence and spectrum of fetal anomalies. Studies conducted in specific geographical regions, therefore, provide valuable insights into local patterns of congenital anomalies and help guide public health interventions. In the sub-Himalayan region of North India, where healthcare resources and access to advanced prenatal diagnostics may vary, such studies are particularly important for improving maternal-fetal healthcare strategies.

The findings of the present study reinforce the importance of multidisciplinary collaboration in the evaluation of fetal anomalies and pregnancy loss. Effective investigation of such cases requires coordination between obstetricians, radiologists, pathologists, geneticists, and laboratory scientists. The integration of clinical data, fetal morphological examination, and molecular genetic testing allows for a more comprehensive understanding of the underlying etiological factors and improves the quality of genetic counselling provided to affected families.

Overall, the results of the present study demonstrate that chromosomal abnormalities contribute significantly to pregnancy loss in the studied population, with autosomal trisomies representing the most common genetic defects. At the same time, the presence of structurally abnormal fetuses with normal chromosomal findings highlights the multifactorial nature of pregnancy loss and underscores the need for comprehensive clinical evaluation.

Further research incorporating larger sample sizes and advanced genomic technologies such as chromosomal microarray analysis and next-generation sequencing may provide deeper insights into the genetic mechanisms underlying fetal anomalies and pregnancy loss. Such approaches may also improve the detection of submicroscopic chromosomal abnormalities and gene-level mutations that cannot be identified using conventional aneuploidy screening techniques.

The present study has certain limitations that should be considered while interpreting the findings. First, the sample size was relatively small (*n *= 39), and the study was conducted at a single tertiary care center, which may limit the generalizability of the results to the broader population. Second, molecular analysis in this study was primarily restricted to the detection of common chromosomal aneuploidies using QF-PCR. Therefore, structural chromosomal rearrangements, microdeletions, microduplications, and other submicroscopic copy number variations could not be identified. Advanced genomic techniques such as chromosomal microarray analysis or next-generation sequencing could potentially improve diagnostic yield in future studies. Additionally, parental karyotyping was not performed, which could have provided further insight into inherited chromosomal abnormalities contributing to pregnancy loss.

## Conclusions

The present study highlights the significant contribution of chromosomal abnormalities to pregnancy loss in the studied population, with autosomal trisomies representing the most common genetic findings. Trisomy 21 was the most frequently detected abnormality, followed by trisomy 18, while other chromosomal abnormalities were identified in a smaller subset of cases. CNS anomalies constituted the most common structural abnormalities observed during fetal examination and were frequently associated with chromosomal defects.

A substantial proportion of cases demonstrated normal chromosomal constitution, indicating that pregnancy loss is often multifactorial and may involve non-genetic or maternal factors. Rapid molecular evaluation using QF-PCR provides an effective diagnostic approach for detecting common aneuploidies in fetal tissue and can significantly aid in etiological diagnosis. Integration of fetal morphological examination with molecular genetic testing and maternal clinical assessment may improve diagnostic accuracy and provide valuable information for genetic counselling and future reproductive planning in affected couples.
